# Mechanism of and Therapeutic Strategy for Atrial Fibrillation Associated with Diabetes Mellitus

**DOI:** 10.1155/2013/209428

**Published:** 2013-03-14

**Authors:** Yubi Lin, Hairui Li, Xianwu Lan, Xianghui Chen, Aidong Zhang, Zicheng Li

**Affiliations:** Department of Cardiology, The First Affiliated Hospital of Jinan University, Guangzhou 0086-510630, China

## Abstract

Diabetes mellitus (DM) is one of the most important risk factors for atrial fibrillation (AF) and is a predictor of stroke and thromboembolism. DM may increase the incidence of AF, and when it is combined with other risk factors, the incidence of stroke and thromboembolism may also be higher; furthermore, hospitalization due to heart failure appears to increase. Maintenance of well-controlled blood glucose and low levels of HbA1c in accordance with guidelines may decrease the incidence of AF. The mechanisms of AF associated with DM are autonomic remodeling, electrical remodeling, structural remodeling, and insulin resistance. Inhibition of the renin-angiotensin system is suggested to be an upstream therapy for this type of AF. Studies have indicated that catheter ablation may be effective for AF associated with DM, restoring sinus rhythm and improving prognosis. Catheter ablation combined with hypoglycemic agents may further increase the rate of maintenance of sinus rhythm and reduce the need for reablation.

## 1. Introduction

Atrial fibrillation (AF) is the most common sustained cardiac rhythm disorder and contributes to thromboembolism. The presence of AF is an independent risk factor for thromboembolism; especially stroke in association with AF increases mortality and morbidity, leading to greater disability, longer hospital stays, and worse quality of life [1, 2]. There are approximately 40 million people with diabetes mellitus (DM) and 10 million with AF in China, and morbidity from AF is increasing with the aging population. AF seriously affects people's health. The abnormal glucose metabolism and DM can facilitate the occurrence and development of AF and are associated with a worse prognosis.

## 2. DM Is a Strong Risk Factor for AF

### 2.1. DM Increases the Incidence of AF

AF commonly coexists with cardiovascular risk factors and disorders, which in turn increase the risk of the complications associated with the arrhythmia. The common risk factors for AF are cardiovascular, including hypertension, congestive heart failure, valvular heart disease, and vascular disease. As a cardiovascular risk factor, DM may be associated with the development and progression of AF. Investigators in the VHAH study reported the incidence of AF in patients with DM to be 14.9%, which was significantly higher than that in patients with hypertension but not DM (*P* < 0.0001). DM is a strong and independent risk factor for the occurrence of AF, with an odds ratio (OR) of 2.13 (*P* < 0.0001) [3]. The VALUE trial showed that new onset DM could be attributed to a significant increase in the new onset of AF (relative risk (RR) 1.49, *P* = 0.0031) and a higher risk of developing persistent AF (RR 1.87, *P* = 0.0014). When patients with new onset DM developed AF, the occurrence of heart failure was much higher (RR 3.56, *P* < 0.0001) [4]. In Nichols' 7.2 ± 2.8-year follow-up study, the age- and gender-adjusted incidence of AF in DM patients (9.1/1000 person-years) was greater than that in non-DM patients (6.6/1000 person-years). Sex is an independent risk factor for AF; women with DM have about a 26% increased risk of developing AF [5]. A meta-analysis [6] of seven prospective cohort studies and four case-control studies included 1,686,097 cases, of which 108,703 had AF. The study indicated that DM was associated with about a 40% increased risk of AF compared with non-DM patients (RR 1.39, *P* < 0.001). After adjusting for multiple risk factors for AF, the RR of AF in patients with DM was still 1.24, and the population-attributable fraction of AF owing to DM was 2.5%. Among 75-year-old patients, occurrence of AF was associated with long-term hyperglycemia, and it was suggested that proactive screening for DM or prediabetes should be performed in patients with a history of AF more than 5 years [7].

### 2.2. DM Increases the Incidence of AF When Accompanied by Other Diseases

DM with hypertension further increased the OR of AF to 3.3, from 0.7 in patients with hypertension only and 2.0 in those with DM only, though this increase had no statistical significance after adjusting for insulin resistance, suggesting that insulin resistance may be the underlying mechanism of AF [8]. The population-attributable fraction of AF owing to metabolic syndrome (hypertension, increased low-density lipoprotein, hypertriglyceridemia, and impaired glucose metabolism) was 22% and the RR was 4.40 in patients with all five components of this syndrome compared with those without the disorder; the more the components, the higher the RR. The multivariate-adjusted RR for impaired glucose tolerance was 1.16. This indicates that the risk of AF increases significantly when DM is combined with other risk factors [9].

### 2.3. Poorly Controlled Blood Glucose Increases the Risk of AF

The risk of AF is increased by 3%/year with prolonged DM. Compared with people without DM, the adjusted ORs for DM patients with average HbA1c ≤ 7, 7-8, 8-9, or >9 were 1.06, 1.48, 1.46, and 1.96, respectively [10], in a prospective cohort study involving 13,025 participants. There was a positive linear association between HbA1c and the risk of AF in patients with or without DM, with hazard ratios of 1.13 and 1.05, respectively. Fasting blood glucose and insulin level were correlated with the risk of AF in DM patients, but not in people without DM. For every 1% increase in HbA1c, the risk of AF increased by 5% in non-DM individuals, but by 13% in those with DM. The risk of AF has also been shown to increase with the duration of DM, high HbA1c levels, and poor blood glucose control [11].

## 3. Prognostic Evaluation of DM Patients with AF

AF is a marker of poor prognosis in patients with DM and increases the risk of cardiovascular events and all-cause mortality. Diabetic neuropathy may mask the cardiac symptoms of first-recorded AF, making it asymptomatic, which may increase the risk of further cardiovascular events and mortality [12]. The ADVANCE study showed a 61% (*P* < 0.0001) increase of multivariate-adjusted all-cause mortality in DM patients with AF during the 4.3-year follow-up period compared with those without AF, and cardiovascular mortality, risk of stroke, and heart failure increased significantly (all *P* < 0.001). Routine treatment with a fixed combination of perindopril and indapamide reduced blood pressure by 5.3/2.3 mmHg and by 5.9/2.3 mmHg more than placebo in patients with AF, significantly decreasing the risk of cardiovascular events and all-cause mortality. Thus, a comprehensive therapeutic strategy that aggressively targets all cardiovascular risk factors is very important for DM patients with AF [13].

DM was an independent risk factor for the recurrence of AF in patients with persistent AF who underwent successful synchronization with current cardioversion, over a median follow-up period of 74 days. In patients with DM, the sinus rhythm maintenance rate was 45.2%, which was lower than that of patients without DM (66.8%, *P* < 0.0001) [14]. A study [15] reported that even after successful direct current cardioversion in DM patients with persistent AF, there was no significant improvement of endothelial responsiveness to supine shear stress or orthostatic modulation, which may be associated with DM-induced endothelial relaxation-constriction dysfunction. In patients with AF alone or in association with hypertension, endothelial dysfunction improved significantly after successful direct current cardioversion. DM-induced oxidative stress injury may be an underlying mechanism of AF [16].

## 4. Underlying Mechanisms of Diabetes-Induced AF

The precise pathophysiologic mechanisms that cause AF in DM patients are unknown [17]. The proposed mechanisms include autonomic remodeling, structural remodeling, electrical remodeling, and insulin resistance. There have been no studies of the role of DM-associated atrial inflammatory reaction and stress reaction in the occurrence of AF.

### 4.1. Autonomic Remodeling

Cardiac autonomic neuropathy (CAN) is a common complication of DM that significantly increases mortality. Reports of the morbidity from CAN in DM patients are inconsistent, which may be related to variations in the populations studied and the methods used to screen for CAN [18]. The morbidity from CAN was low (2.5%) in patients undergoing primary prevention, but very high (90%) in those with long-term type 1 DM awaiting pancreas transplantation [19, 20]. Based on heart rate variability (HRV) tests and spectral analysis of RR intervals, the morbidity from CAN in type 2 DM (T2DM) was 34.3%. Age, sex, and other risk factors may be associated with the progression of CAN [21]. A recent report showed that CAN was common in patients with T2DM, with a morbidity of 44.3%; CAN was associated with modifiable factors such as carotid lipid deposition, hypertension, dyslipidemia, smoking, poorly controlled glycemia, and microvasculopathy. Management of these risk factors can prevent the occurrence and progression of CAN [22], which is closely related to the effects of risk factors such as poor glycemic control, a history of hyperglycemia, age-related neural injury, and high blood pressure. Hyperglycemia plays an important role in the pathogenesis of CAN by impairing nerve blood perfusion and activating cellular metabolism and redox-associated biologic pathways.

The autonomic dysfunction in DM patients can be caused by hyperglycemia-related pathophysiologic pathways such as the formation of advanced glycation end products, elevated oxidative/nitrosative stress with increased production of free radicals, and activation of the polyol and protein kinase C pathway, as well as poly-ADP ribosylation and neuronal damage-associated genes [18]. Most DM patients have autonomic imbalance—that is, enhanced sympathetic activity and decreased parasympathetic activity—regardless of whether they have DM neuropathy. Well-controlled blood glucose and use of angiotensin converting enzyme inhibitors have a beneficial effect on HRV [23]. A DM rat model established by intravenous injection of streptozotocin indicated that sympathetic stimulation increased the incidence of AF in DM rats but not in controls (*P* < 0.01). Sympathetic stimulation significantly shortened the effective refractory period (ERP) of atrial cells in both groups, but the heterogeneity of the atrial ERP increased in the DM rats only. Parasympathetic stimulation also increased the incidence of AF via shortening of the atrial ERP in both DM rats and controls. Immunohistochemical staining of the right atrium aimed at determining the distribution of sympathetic nerves revealed that tyrosine hydroxylase positive nerves were significantly more heterogeneous in DM rats than in control rats, whereas the heterogeneity of acetylcholine esterase positive nerves did not differ between the two groups. This evidence indicates that cardiac adrenergic nerve stimulation in individuals with DM can lead to AF. The heterogeneity of sympathetic innervation is increased in DM, which suggests that autonomic remodeling may increase susceptibility to AF in this group [24]. Animal studies have indicated that sympathetic nerve remodeling may play an important role in the development and progression of AF in DM patients.

### 4.2. Electrical and Structural Remodeling

DM cardiomyopathy (i.e., DM-related cardiac structural and functional changes that are not caused by coronary atherosclerosis or hypertension) can lead to left ventricular hypertrophy and increased susceptibility to ischemic injury and the morbidity of heart failure, thereby increasing mortality. Possible pathophysiologic mechanisms of DM cardiomyopathy include myocardial hypertrophy, myocardial lipotoxicity, oxidative stress, cellular apoptosis, interstitial fibrosis, contraction-relaxation dysfunction, impaired myocardial contractile reserve, mitochondrial dysfunction, and disorders of myocardial metabolism [25]. A study employing the genetic T2DM (Goto-Kakizaki) rat model found no difference in ERP between DM and control rats, but the intra-atrial activation time of the DM group was much longer. In a DM rat model, a single premature electrical stimulation induced large numbers of repetitive atrial responses [26]. During catheter ablation, using a three-dimensional mapping system, the activation time of both atria was significantly longer, and the bipolar voltage significantly decreased in a DM group [27]. Maximal P-P interval and the degree of P wave dispersion were significantly increased in prediabetic patients without a history of coronary heart disease, hypertension, or left ventricular hypertrophy, and the latter was positively related to fasting blood glucose level [28].

Atrial fibrosis is also an important mechanism for cardiac structural and electrical remodeling. In a DM rat model, there are widespread fibrotic deposits in the atria that are prone to the formation of anchoring points for reentry circuits and changes in the forward propagation of fibrillatory wavelets and thus cause atrial fractionated potentials and conduction delay [26]. Oxidative stress may also be involved in the formation of hyperglycemia-associated AF substrates, leading to atrial fibrosis. A study in which atrial tissue was collected from DM patients during coronary artery bypass graft surgery found that mitochondrial dysfunction in the atrial myocardium can cause excessive oxidative stress [29]. In DM rats, the expression of connexin-43 was elevated, whereas its phosphorylation was decreased in the atrial myocardium, which leads to disorders of intercellular electrical coupling and atrial arrhythmia [30]. Advanced glycation end products (AGEs) and AGE receptors (RAGEs) (the AGE-RAGE system) mediate the diffuse interstitial fibrosis of the atrial myocardium in DM rats through upregulation of the expression of growth factors by connective tissue, and cause structural remodeling. AGE inhibitors can downregulate the expression of growth factors and significantly inhibit the progression of DM-induced atrial fibrosis [31]. AGE inhibitors may be used in the upstream therapy of DM-related fibrosis in the future.

### 4.3. Insulin Resistance

Previous studies have demonstrated that patients with insulin-dependent DM suffer AF attacks while hypoglycemic. Some experts consider the cause of such AF attacks to be fluctuations in glycemia rather than the hyperglycemic state itself [17]. A study has found that blood glucose is significantly elevated during an AF attack and that a high dose of insulin (10 times the daily dose) is required to control it. After successful cardioversion of AF, the insulin dose required is significantly decreased [32].

## 5. Therapeutic Strategy

### 5.1. Anticoagulation Therapy

AF is a major risk factor for stroke and thromboembolism. The mortality, disability rate, and risk of stroke recurrence are all higher in stroke caused by AF than in that due to other causes. The risk of stroke varies among DM patients with AF, and treatment relies on risk evaluation and appropriate anticoagulation therapy. The simplest way to assess the risk of stroke is the CHADS_2_ scoring system. The European Society of Cardiology Guidelines for the Management of Atrial Fibrillation (2010 ESC) recommended the use of a more appropriate system, the CHA_2_DS_2_-VASc. In AF patients with DM, if the risk of major bleeding is low or the benefit/risk ratio is considered high, an oral anticoagulant such as a vitamin K antagonist (VKAs) is the first choice independent of rate control or rhythm control. The dose of VKAs depends on the target intensity INR (International Normalized Ratio) of 2.0–3.0, with a target value of 2.5. New anticoagulants (e.g., Dabigatran etexilate, Apixaban) are alternatives to VKAs [33].

### 5.2. Upstream Therapy

Atrial structural remodeling or fibrosis can decrease the conduction speed of the atrial myocardium, which may be the underlying mechanism of DM-related AF. Angiotensin II receptor blockers (ARBs) may play an important role in the upstream therapy of AF through the following mechanisms: decreasing atrial fibrosis and delaying atrial structural remodeling; inhibiting the production and facilitating the degradation of collagen fibers, thereby decreasing the left atrial overload that is the major reason for the development and progression of AF [26]; modifying the potassium channel current and downregulating the Ito current, the latter of which plays an important role in atrial electrical remodeling [34]; exerting various effects on the subunits of the potassium channel (e.g., losartan blocks the HERG, KvLQT1, mink, and hKv1.5 subunits) [35]; regulating L-type and T-type calcium channels and inhibiting the progression of AF substrates [36, 37]; prolonging the ERP and increasing the frequency adaptability of atria; and decreasing the hyperactivity of the sympathetic nerve system caused by angiotensin II [38]. In hypertensive DM patients with paroxysmal AF, the blood pressure lowering effect is similar in both groups. In patients treated with valsartan/amlodipine, the rate of recurrence of AF is significantly decreased compared with that in patients treated with atenolol/amlodipine, especially those given amiodarone or propafenone compared with those who take other or no antiarrhythmic agents [39].

Several studies indicated thiazolidinedione as a class of peroxisome proliferator-activated receptor-*γ* activator, may potentially benefit for AF prevention [40–42], especially pioglitazone, due to attenuate atrial fibrosis, inflammation activation, oxidative stress, and apoptosis in the atrium of experiment models [43–47]. The thiazolidinedione may be a novel upstream therapy for AF in DM patients, but still needs further large-scale randomized, controlled trials with long-term follow-up period to evaluate the potential role of AF prevention [48].

### 5.3. Antiarrhythmic Therapy and Catheter Ablation

Antiarrhythmic treatment in AF patients includes rate control and rhythm control; the choice of drug should be individualized. Commonly used antiarrhythmic drugs that control heart rate in AF patients are *β*-blockers, nondihydropyridine calcium channel blockers, and digitalis glycosides. Drugs commonly used to control heart rhythm are amiodarone and dronedarone. After treatment with the full dose of an antiarrhythmic drug, catheter ablation may be of further benefit in the treatment of AF [33]. A large prospective study enrolled 263 patients receiving first-time ablation for encircling pulmonary veins guided by the CARTO system; 31 of these had DM. Although the age, duration of AF, left atrial size, incidence of hypertension, and proportion with underlying heart disease were significantly higher in the DM patients compared with the non-DM patients, the rate of recurrence of AF after first-time ablation was similar in the two groups (32.3% versus 22.4%, *P* = 0.24). The possibility of an episode of asymptomatic AF caused by DM neuropathy cannot be excluded in this study, so the success rate of the AF ablation may have been overestimated. This study also suggested that the incidence of procedure-associated complications was higher in the DM patients (29% versus 8.2%, *P* = 0.002). The major complications were hemorrhage and thromboembolism. When hemorrhage was excluded, the incidence of complications was 6.4%. There was an increased tendency to develop cardiac tamponade and thromboembolism in the DM patients; possible reasons include the following: AF is a risk factor for cerebral embolism; DM can cause both prothrombotic and proinflammatory states; and hyperinsulinemia can induce a prothrombotic state that is closely related to metabolic disorders (postprandial hyperglycemia, high levels of free fatty acids, and hypertriglyceridemia) involving platelets, the coagulation cascade, and fibrinolysis [49]. Another study included 70 DM patients with AF (including paroxysmal and persistent AF) and assigned catheter ablation (one ablation procedure without drug treatment) or antiarrhythmic agents randomly. After 1 year of followup, sinus rhythm was maintained in 42.3% and 80% of patients treated with drugs or catheter ablation, respectively (*P* = 0.001). The Life Quality Scale score was significantly higher in the ablation group than in the drug group; 17.1% of patients developed obvious drug-associated side effects, and the hospitalization rate was significantly higher in the drug treatment group (*P* = 0.01). The major complication of catheter ablation was puncturing site bleeding. This evidence suggests that catheter ablation can significantly improve the prognosis of DM patients with AF compared with drug treatment alone [50]. Assessment of DM patients with AF 18.8 ± 6.4 months after catheter ablation showed that, though the recurrence rate was higher in patients with glucose metabolism derangement than in those without DM (*P* = 0.02), it still reached 18.5%, which may be associated with intra-atrial conduction delay and decreased voltage [27].

Pioglitazone is a commonly prescribed drug in clinical practice with anti-inflammatory and antioxidative effects. In a contrast study, 150 selected T2DM patients with paroxysmal AF were divided into two groups depending on whether pioglitazone was given after catheter ablation. Antiarrhythmic drugs were discontinued in patients who remained in sinus rhythm. After an average 15-month followup, compared with the control group, the rate of maintenance of sinus rhythm was higher (86.3% versus 70.7%, *P* = 0.03) and the proportion that underwent a second ablation was lower (9.8% versus 24.2%, *P* = 0.03) among patients treated with pioglitazone. This evidence indicates that, in T2DM with paroxysmal AF, pioglitazone can significantly increase the success rate of catheter ablation and decrease the recurrence of AF. Left atrial size is associated with recurrence of atrial arrhythmia, which can be decreased by both pioglitazone and ARBs [51].

## 6. Conclusion

DM is one of the most important risk factors for the development and progression of AF and can predict the occurrence of stroke and thromboembolism. Long-term anticoagulation therapy with VKAs is recommended in AF patients with DM. DM can increase the incidence of AF, and when it is combined with other risk factors, the incidence of stroke and thromboembolism may be even higher. The incidence and rate of hospitalization due to heart failure may be increased. Maintenance of well-controlled blood glucose and low HbA1c according to guidelines may decrease the incidence of AF. The mechanisms of AF associated with DM are autonomic remodeling, electrical remodeling, structural remodeling, and insulin resistance; there are few studies concerning the role of inflammation and oxidative stress in the pathogenesis of AF. Inhibition of the renin-angiotensin system is expected to be an upstream therapy for this type of AF. Some studies have indicated that catheter ablation may be effective in AF associated with DM, restoring sinus rhythm and improving prognosis. Catheter ablation combined with hypoglycemic agents may further increase the rate of maintenance of sinus rhythm and also reduce the risk of need for reablation.
